# Transcatheter Pulmonary Valve Outcomes and Mechanisms of Dysfunction

**DOI:** 10.3390/jcm15010213

**Published:** 2025-12-27

**Authors:** Amr Matoq, Shabana Shahanavaz

**Affiliations:** 1 The Heart Institute, Cincinnati Children’s, 3333 Burnet Avenue, MLC 2003, Cincinnati, OH 45229, USA; 2 Department of Pediatrics, University of Cincinnati College of Medicine, Cincinnati, OH 45229, USA

**Keywords:** transcatheter pulmonary valve (TPV), right ventricular outflow tract (RVOT), transcatheter pulmonary valve replacement (TPVR), hypo-attenuated leaflet thickening (HALT), Reduced leaflet motion (RELM), Patient–Prosthesis Mismatch (PPM)

## Abstract

Since the initial use of transcatheter pulmonary valves (TPVs), various generations of balloon-expandable and self-expanding valves have become available to effectively treat dysfunctional right ventricular outflow tract (RVOT), providing a less invasive alternative to surgery. In this review, we summarize the most up-to-date TPVR outcomes and mechanisms of TPV dysfunction.

## 1. Introduction

**Figure 1:**
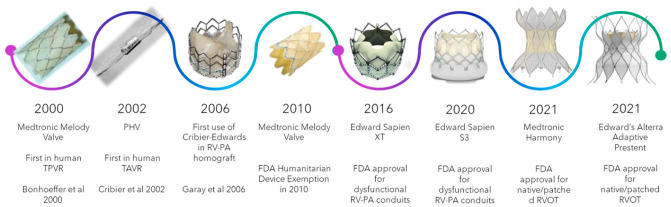
Timeline of transcatheter valves over the last 2 decades.

Since their first use in humans [1], transcatheter pulmonary valves (TPVs) have been widely used in patients with congenital heart disease. Several versions of TPVs have been introduced and have successfully provided an excellent alternative to surgical pulmonary valve replacement (SPVR), minimizing the number of sternotomies that are destined to occur over the lifetime of patients with tetralogy of Fallot (TOF), pulmonary stenosis (PS), and other conotruncal anomalies with dysfunctional right ventricular outflow tracts (RVOTs). Over the last two decades, numerous designs of TPVs have been developed and used in the United States and other parts of the world (Figure 1). Due to the availability of wide variety of TPV platforms, treating dysfunctional RVOTs with variable anatomy has become achievable through transcatheter means, resulting in outcomes that are comparable to SPVR [2]. Like all bioprosthetic valves (BPV), TPV dysfunction is inevitable. In this review, we summarize the outcomes of TPVs, the mechanisms of failure, and management of dysfunctional TPVs.

## 2. Indications and Technical Considerations

Surgical or transcatheter intervention is indicated for symptomatic patients with moderate or greater RVOT obstruction (peak velocity more than 3–4 m/s, or peak gradient 36–64 mmHg [3]), asymptomatic patients with severe obstruction (peak velocity more than 4 m/s, peak gradient > 64 mmHg, or mean gradient > 35 mmHg), or right ventricular systolic pressure greater than 2/3 systemic [4]. In patients with pulmonary regurgitation (PR), intervention is indicated if symptomatic with moderate or severe PR [3,4]. PVR in symptomatic patients is associated with improvement in symptoms and functional class [5]. In asymptomatic patients, MRI-derived RV volume criteria can help in guiding timing of intervention. Studies have shown reduction of RV volume when PVR is performed at 150–160 mL/m [2,6,7,8]. It is worth noting that majority of these data are derived from adult populations. Both the American Heart Association (AHA) and European Society of Cardiology (ESC) provide criteria for intervention in asymptomatic adult patients with moderate or severe PR that evaluates RV volume, RV and LV systolic function, and progressive decline in objective exercise capacity [3,4]. While AHA does not specify the type of intervention (surgical vs. transcatheter), ESC recommends a transcatheter approach if anatomically suitable and in the appropriate substrate [4].

There are several technical considerations that affect the choice of TPV, procedural planning, and follow-up: first, the landing zone for TPV. RV-PA conduits are often stenotic and require conduit preparation prior to valve implantation to achieve a large diameter. A RV-PA conduit can be dilated beyond its nominal diameter [9,10]. Pre-stenting of conduits prior to melody valve implantation has been shown to reduce valve re-interventions [11,12]. The use of covered stents is effective in reducing conduit tears [13] and is an important step in conduit preparation, especially if dilating a calcified conduit beyond its normal diameter. Dysfunctional bioprosthetic valves (BPVs) are often a suitable landing zone for TPVs (valve in valve), with excellent reported outcomes [14]. Except for few, most BPVs can be dilated or fractured to accommodate larger valves in the valve [15,16]. Native RVOT represents a challenge for balloon-expandable valves given its larger diameters and systolic/diastolic variation in diameter [17]. Pre-stenting provides a secure landing zone but can interfere with advancing the valve system [18]. Pre-stenting is necessary with melody valves given the risk of valve stent fracture and dysfunction [12].

Another technical consideration is coronary compression. Balloon compression testing is an important step prior to TPV implantation. CT scans can predict the risk of coronary compression to some extent [19,20] but do not negate the need for balloon compression testing. The incidence of coronary compression with TPV is only 4–6% [21,22], but it has serious implications and may necessitate urgent surgery [23]. Incidence of compression is higher with coronary anomalies and post-Ross procedure [21].

Another consideration is ventricular arrhythmia. Most patients undergoing TPV are at risk for ventricular arrhythmia. TPV can cover critical areas of the infundibular septum and render it inaccessible for future electrophysiological studies (EPSs) [24]. Those at risk may need to undergo EPS prior to TPV. Also, in balloon-expandable valves and pre-stent, the interaction of the proximal part of the valve with RVOT leads to ventricular arrhythmia [25,26], most of which was non-sustained [27].

## 3. Types of Transcatheter Pulmonary Valves

Currently, there are two main types of transcatheter valves: first, balloon-expandable valves, including Medtronic Melody valve (Medtronic, Minneapolis, MN, USA) and Sapien transcatheter heart valve (Edwards Lifesciences, Irvine, CA, USA). Another balloon-expandable valve is Myval THV (Meril Life Sciences Pvt Ltd., Vapi, Gujarat, India), which is not currently available in the United States. Those valves need a suitable landing zone like an RV-PA conduit, bioprosthetic valve, or native RVOT with a suitable diameter. The second valve type is self-expanding valves and pre-stent. These are designed to fit native dysfunctional RVOT and have a larger diameter and longer frame. Self-expanding valve platforms have provided an alternative to patients with larger dysfunctional native RVOT, in whom balloon expandable valves were not suitable.

### 3.1. Transcatheter Balloon-Expandable Valves

There are two balloon-expandable valves that are available in the United States. The first reported use of any TPV in humans was in 2000 using the Medtronic Melody valve (Medtronic, Minneapolis, MN, USA) [1]. It is made of a bovine jugular vein sewn into a Cheatham-Platinum stent (CP stent) and is delivered via the Medtronic Ensemble II delivery system, which comes in three sizes (18, 20, and 22 mm) for valve sizes of 20 mm and 22 mm. The maximum diameter is 22 mm, but overexpansion to 24 mm has been successfully reported [28]. The Melody valve Ensemble covers the valve until it reaches the deployment site, which improves trackability and potentially helps to avoid tricuspid valve injury. The Melody valve received Food and Drug Administration (FDA) approval under the Humanitarian Device Exemption (HDE) in 2010 and is currently approved for dysfunctional RV-PA conduits and BPVs.

The first version of the Edwards Sapien transcatheter heart valve (Cribier-Edwards) was used in an RV-PA homograft in 2006 [29]. Subsequently, the Sapien XT and S3 were developed. The valve is made of bovine pericardium, chemically treated and sewn into a cobalt-chromium stainless steel stent. The Sapien S3 is available in 20, 23, 26, and 29 mm. It was originally designed for delivery into the aortic position with the low-profile Commander delivery system through a 14–16 Fr expandable sheath (eSheath) [30]. To achieve this low profile, the valve is crimped on the balloon’s shaft and then advanced over the balloon in vivo. When used in the pulmonary position, the use of a large delivery sheath is not prohibitive and is preferred over advancing the uncovered valve through the tricuspid valve. Therefore, a 65 cm long Gore Dry-Seal sheath to deliver the valve has been utilized, and it has been shown to minimize tricuspid valve injury [31,32]. Sapien XT received FDA approval for placement in dysfunctional RV-PA conduits in 2016, and the S3 received FDA approval in 2020. The newest version, the Sapien 3 Ultra Resilia, has an anticalcification treatment on the leaflets and a longer skirt to prevent paravalvular leak. A clinical trial is currently taking place to expand FDA approval to the pulmonic position (NCT02744677).

Other balloon-expandable valves include the Myval THV (Meril Life Sciences Pvt Ltd., Vapi, Gujarat, India), which was approved in India in 2018 and received the CE mark in Europe in 2019 for transcatheter aortic valve replacement (TAVR) [33]. It is made of bovine pericardial leaflets sewn into a nickel–cobalt stent frame. The stent frame has a covered proximal half and a bare-metal distal half. The valve diameter goes up to 32 mm [34].

### 3.2. Outcomes of Balloon-Expandable Valves

Long-term outcomes of the Melody TPV Investigational Device Exemption (IDE) trial showed a 10-year survival of 90% for the entire cohort (n = 149). Freedom from reoperation was 79%, freedom from any reintervention was 60%, and freedom from valve dysfunction was 53%. Freedom from TPV infective endocarditis (IE) at 10 years was 81% [35]. In another study comparing the Melody valve to SPVR, there was no statistically significant difference in 10-year survival (94% in the Melody group; n = 241 versus 92% in the SPVR group; n = 211) [2].

Long-term outcomes from the COMPASSION (Congenital Multicenter Trial of Pulmonic Valve Regurgitation Studying the SAPIEN Transcatheter Heart Valve) trial are not yet available. Three-year follow-up showed a survival rate of 98% (n = 69). Freedom from reintervention after three years was 93.7%, and freedom from IE after three years was 97.1% [36]. One-year follow-up for COMPASSION S3 (for the Sapien S3 THV valve) showed no mortality, no reintervention, and only 4.3% valve dysfunction (n = 57) [37].

Multicenter data from registries and retrospective single-center studies have outlined excellent medium-term outcomes for valve performance and freedom from reintervention. In a large multicenter registry with a total of 2476 patients who underwent transcatheter pulmonary valve replacement (TPVR) with both Melody (82%) and Sapien (18%), the cumulative incidence of death eight years post-TPVR was 8.9%. The cumulative incidence of any reintervention at eight years was 25%, and surgical reintervention was 14.4% [38].

### 3.3. Self-Expandable Platforms

Medtronic Harmony (Medtronic, Minneapolis, MN, USA) was the first self-expandable valve to receive FDA approval for native/patched RVOT in 2021. The valve frame is made of nitinol, covered with polyester fabric along its entire length, and within the frame is a treated porcine pericardium valve. It is available in two sizes, TPV 22 and TPV 25, with frame lengths of 51–55 mm, inflow diameters of 41–54 mm, and outflow diameters of 32–43 mm [17].

The Alterra Adaptive Prestent (Edwards Lifesciences, Irvine, CA, USA) was introduced to create a landing zone in native/patched RVOT for the 29 mm Sapien S3. First in-human implantation of the Alterra pre-stent was in 2018 [39], and it received FDA approval in 2021. The frame is made of nitinol, partially covered with PTFE, with a length of 49 mm and an inflow/outflow diameter of 40 mm. The waist of the stent represents a rigid landing zone for the 29 mm Sapien S3.

The Venus P-valve (Venus MedTech, Hangzhou, China) is a porcine pericardial valve within a nitinol frame that is covered with pericardial tissue except the distal end. It is available in 18 mm to 36 mm diameter, and 20 mm to 35 mm length [40]. The valve has been utilized extensively in Aisa and Europe, and a pivotal trial is currently undergoing in the United States (NCT06010563).

Other platforms of self-expanding valves are available outside the US, including the Med-Zenith PT-valve (Med-Zenith, Beijing, China) and the Pulsta valve (TaeWoong Medical, Gyeonggi-do, Republic of Korea). All three valves have self-expanding nitinol frames, covered (partially or completely) with pericardium, and they house a porcine pericardial valve [40,41,42].

### 3.4. Outcomes of Self-Expandable Valves

The five-year outcome from the early feasibility study for Harmony showed no procedural mortality. There was one mortality after three years of follow-up, with no clear relationship to the valve. Two patients underwent explantation, and two patients underwent catheter-based intervention (TPV-in-TPV) [43].

A recent multicenter registry study [44], which included patients who received Harmony TPV 22 and TPV 25 (excluding those enrolled in the post-approval study), showed a procedural success rate of 99.6% (n = 243) and a serious adverse event rate of 4.1%. At a median follow-up of 13 months, 98% had mild or less pulmonary regurgitation (PR). At one year, freedom from mortality (all-cause) was 98%, freedom from infective endocarditis (suspected or proven) was 98%, and freedom from RVOT reintervention was 99% [44].

The Alterra Adaptive Prestent multicenter pivotal study showed 97% successful implantation (n = 60). At two years, 92.5% had mild or less PR, and there was no endocarditis, mortality, or explantation [45]. One patient underwent transcatheter reintervention for RVOT obstruction [45], and one patient had pericarditis with effusion, which was managed medically [25].

As for the Venus *p*-valve, a recent 3-year CE study included 81 patients and reported no mortality. There are fewer incidences of VT compared to other self-expanding platforms. Infective endocarditis was reported in one patient, and valve thrombosis in one patient, both treated medically [46].

## 4. Mechanisms and Predictors of TPV Dysfunction

**Figure 2:**
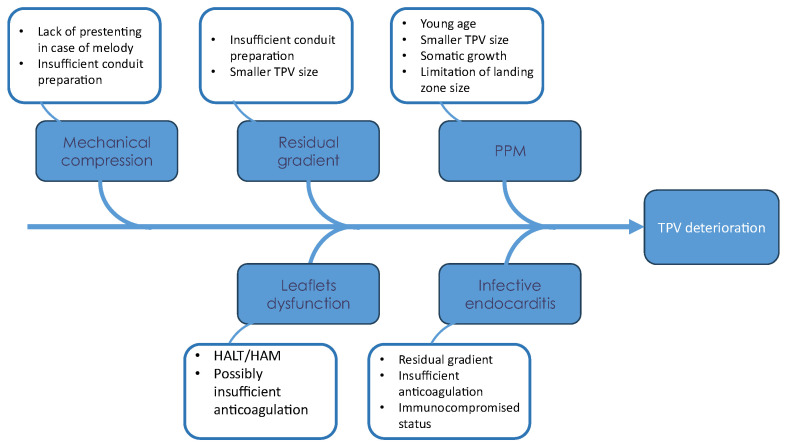
Fishbone diagram for factors associated with TPV deterioration.

Both surgical and transcatheter valves have limited lifespans before valve dysfunction occurs, with variable longevity related to many factors that are summarized in Figure 2. 

### 4.1. Age, TPV Size, and Patient Prosthesis Mismatch (PPM)

Implanting TPV at a young age has been established as a risk factor for reinterventions [35,38,47,48], the majority of which are due to RVOT obstruction [38]. It has also been shown that young age is a risk factor for reintervention due to RVOT obstruction unrelated to endocarditis or valve stent fracture [47]. This can be explained by somatic growth, the development of Patient–Prosthesis Mismatch (PPM), and the limitation of valve size that can be implanted in a child. Smaller TPV size was also identified as a risk factor for reinterventions [48], which is often the case in smaller children with smaller conduits/homografts. However, there is enough evidence that TPV implantation in small children is feasible and provides hemodynamic improvements comparable to surgical valves [49,50]. It can also minimize the overall number of sternotomies for patients and allow them to avoid surgery until they reach an adequate age/size for an adult-sized surgical valve.

### 4.2. Residual Gradient

Immediate post-implant gradient has been proven to be a risk factor for both surgical and transcatheter reintervention [38,51,52]. A residual gradient of less than 15 mmHg has been associated with fewer interventions [52]. There are multiple technical considerations that can help in achieving low residual gradient. In case of conduit and homograft, pre-stenting before implanting the valve can reduce reintervention [11]. In case of valve-in-valve implantation, fracturing the existing bioprosthetic valve to achieve a larger-diameter landing zone is associated with lower residual gradient [15]. In small children, the use of jugular venous access can allow a larger sheath size for larger valves [53,54]. The presence of a higher pre-implantation mean Doppler gradient and RVOT obstruction (as opposed to PR) as an indication for TPVR has been associated with a higher gradient at discharge following TPVR [51].

### 4.3. Mechanical Compression

Melody valve stent fracture and subsequent valve dysfunction were noted in earlier studies and have been significantly reduced by prestentin [11,35,38,51]. Now, pre-stenting and conduit rehabilitation/preparation have become an integral part of the TPVR procedure. Since homografts and bovine jugular conduits can be dilated beyond their nominal diameter [9,10,55], this further highlights the importance of pre-stenting and conduit preparation to achieve a larger landing zone for the TPV.

Stent frame fracture has also been noted in self-expanding platforms, though its clinical impact on valve function and longevity is not yet clear. Stent frame fracture resulting in hemodynamic sequelae has been reported, though it remains very rare (a single case in a cohort of 233) in a large multicenter registry for the Harmony valve [44]. In Alterra pre-stents, stent fracture was reported in 14 patients (n = 60), none of whom required reintervention [45]. Further follow-up may reveal the clinical importance of these noted fractures.

### 4.4. Infective Endocarditis (IE)

Infective endocarditis (IE) is an important risk for all pulmonary valves, whether placed surgically or via a transcatheter approach. The estimated annualized incidence of IE in balloon-expandable valves ranges from 1.95% to 3.6% per patient-year, with 5-year freedom from IE reported between 85.2% and 89% [56,57,58]. While rare, mortality rates from IE are not insignificant, estimated at 6.6% to 14% [57,58]. Management of IE in patients with TPV varies between centers. Transcatheter re-intervention for IE (balloon dilation or valve in valve) has been reported with limited efficacy, with frequent surgical explantation at variable intervals following re-intervention [59].

Risk factors for IE following TPVR include younger age, residual gradient > 15 mmHg, immunocompromised status, previous history of IE [56,57,60,61], and possibly inadequate anticoagulation [58]. In a large retrospective cohort (n = 2476), there was no statistically significant difference in the incidence of IE between different TPV platforms [57]. There are no available prospective studies comparing the incidence of IE between surgical and transcatheter valves, and retrospective results have been variable [2,62].

Limited long-term data are available for self-expanding platforms compared to balloon-expandable valves. For the Harmony TPV, there were no reports of IE in the early feasibility study at 5 years [43]. In a large multicenter registry for Harmony TPV, freedom from IE (suspected or proven) was 98% at 1 year [44]. In the multicenter pivotal study for the Alterra Adaptive Prestent, there were no reports of IE at 2 years [45].

### 4.5. Thrombosis and Leaflet Dysfunction

Subclinical leaflet thrombosis has been studied in bioprosthetic aortic valve (both surgical and transcatheter) and is linked to valve deterioration, stroke, and transient ischemic attack (TIA) [63,64,65]. Early Hypo-Attenuated Leaflet Thickening (HALT) has been reported in 10% of TAVR patients and was demonstrated to be reversible with anticoagulation [64]. Reduced leaflet motion (RELM) was reported in 40% of patients following TAVR, with improvement in leaflet mobility after anticoagulation but no significant change in clinical outcomes [63].

There is limited evidence regarding the incidence and implications of subclinical leaflet thrombosis in TPVR. In a prospective study where a CT scan was obtained at 1 month post-TPVR, incidence of HALT was 58% and RELM 17% (n = 12) [66]. Of these, only one patient had increased RVOT gradient on echocardiogram. In another study, where a CT scan was obtained at median of 8,4 months, the incidence of HALT following TPVR was 17.4% (n = 46), with an increased incidence of valve dysfunction compared to the total cohort [67]. Anticoagulation (warfarin or direct oral anticoagulants [DOACs]) has been reported to reverse HALT in the aortic valve population [63,64]. This might also apply to TPVR patients, although adequate evidence is lacking. Until further data is available, empiric evidence supports screening for HALT in patients with TPVR if valve deterioration is detected on echocardiography. If HALT or RELM is detected, the addition of anticoagulation is suggested. Spontaneous resolution of HALT has also been reported [67].

## 5. Learning Curve with TPVR

The technical challenges associated with transcatheter pulmonary valve replacement (TPVR) are multifactorial and depend on both the underlying anatomical substrate and the specific valve platform employed. The initial learning curve with the Melody valve primarily centered on conduit rehabilitation, which included pre-stenting strategies, coronary compression testing, and the management of conduit rupture. With the transition to bioprosthetic surgical valves, operators developed additional expertise, particularly in valve-fracture techniques performed prior to TPVR to facilitate optimal expansion and hemodynamic results. Across both subsets, the recognition of the importance of complete gradient relief emerged as a critical factor in minimizing valve dysfunction and reducing the risk of infective endocarditis.

The introduction of the Sapien valve, originally designed for transcatheter aortic valve replacement (TAVR), presented unique procedural challenges when adapted for right-sided use. Early experience highlighted the risk of tricuspid valve injury due to the manipulation of a delivery system not specifically engineered for the pulmonary position. This experience subsequently led to the adoption of long Gore DrySeal sheaths, which enhanced control and safety during valve deployment.

**Figure 3:**
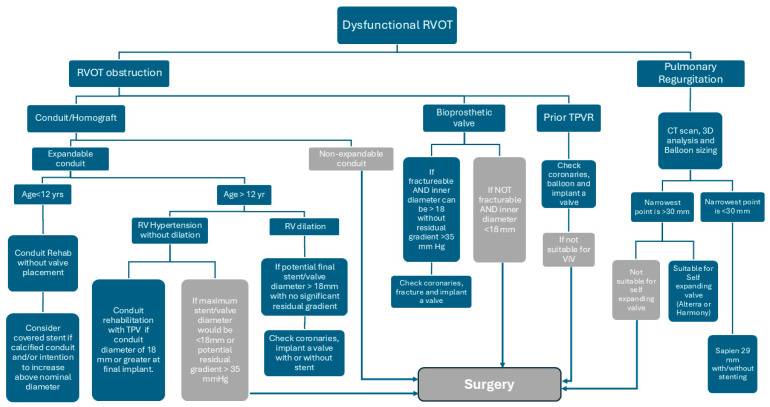
Diagram of our management approach for dysfunctional RVOT.

In patients with a native right ventricular outflow tract (RVOT), the initial approach to balloon-expandable valves mirrored principles established in conduit-based TPVR, emphasizing the role of pre-stenting. However, with advancements in the Sapien platform and increasing operator experience, practice evolved toward direct valve implantation in appropriately selected anatomies. The advent of self-expanding valve platforms introduced a distinct set of considerations, particularly related to anatomical suitability and device–host interaction. The current learning curve continues to evolve, with a growing emphasis on accurate valve sizing using perimeter-derived measurements and a refined understanding of how different valve designs conform to the varied geometries of the native RVOT. We have summarized our approach for managing dysfunction RVOT in Figure 3.

## 6. Conclusions and Future Directions

Great strides have been made in transcatheter valves over the last two decades, providing a comparable alternative to surgical intervention for patients with congenital heart disease. Future studies should focus on long-term follow-up data, which will require collaboration through registries and data sharing. More studies are needed to identify interventions that can help improve valve longevity and alter the risk of valve dysfunction, endocarditis, and thrombosis.

The next phase of transcatheter pulmonary valve (TPV) therapy will focus on improving durability, procedural precision, and integration with multidisciplinary care. Advances in polymeric leaflet materials and anticalcification treatments promise to extend valve longevity, while expandable and re-dilatable valve platforms may accommodate somatic growth and evolving RVOT anatomy. Emerging 3D imaging, computational modeling, and printing technologies will enhance pre-procedural planning by enabling accurate assessment of RVOT geometry, coronary proximity, and landing zone suitability. These tools will also facilitate patient-specific valve design and simulation of procedural outcomes.

Collaboration between interventional and electrophysiology teams will become increasingly important, as many patients remain at risk for ventricular arrhythmias. Integration of electrophysiologic mapping into procedural planning and the development of valve systems that preserve or permit access to arrhythmogenic substrates may improve long-term rhythm management. Finally, ongoing efforts to reduce infective endocarditis through antimicrobial coatings, expand global access, and establish long-term registries will continue to advance the safety, efficacy, and reach of TPV therapy.

## Data Availability

No new data were created or analyzed in this study.
